# Integrating rare and common variation in epilepsy genetics: from genetic architecture to penetrance and clinical expressivity

**DOI:** 10.3389/fgene.2026.1902275

**Published:** 2026-08-24

**Authors:** Ming-Shan Cai, Hou-Rui Li, Peng Liao, Zhen-Liang Hu, Yong-Jun Chen

**Affiliations:** 1 Department of Neurology, The Affiliated Nanhua Hospital, Hengyang Medical School, University of South China, Hengyang, China; 2 Clinical Research Institute, The Affiliated Nanhua Hospital, Hengyang Medical School, University of South China, Hengyang, China

**Keywords:** common variants, epilepsy, penetrance, phenotypic heterogeneity, polygenic risk score, rare variants

## Abstract

Epilepsy genetics has often been interpreted through a useful but simplified dichotomous framework in which severe epilepsies, particularly developmental and epileptic encephalopathies, are attributed mainly to rare, high-effect variants, whereas more common epilepsies are viewed as arising largely from the cumulative effects of common, small-effect variation. Although this framework has been instrumental for gene discovery, molecular diagnosis, and mechanism-based treatment, it does not fully explain incomplete penetrance, intrafamilial phenotypic heterogeneity, or marked differences in severity among individuals sharing the same molecular diagnosis. Evidence from exome sequencing, copy number variant (CNV) studies, and genome-wide association studies increasingly suggests that rare SNVs/indels, CNVs, and common variant should not be interpreted as entirely independent risk sources, but may partially converge on shared genes, pathways, cell types, and neurobiological processes relevant to neuronal excitability, network stability, and seizure susceptibility. Here, we review evidence across epilepsy subtypes, focusing on convergence and divergence across the allelic spectrum, and discuss how polygenic background and other modifiers may influence penetrance and clinical expressivity among carriers of rare pathogenic variants and CNVs. We also consider implications for variant interpretation, genetic counseling, risk stratification, and precision medicine, while emphasizing that most rare–common integrated models remain insufficiently validated for routine clinical decision-making.

## Introduction

Epilepsy comprises a clinically and genetically heterogeneous group of neurological disorders (Fisher et al., 2014; Zhang et al., 2024), yet its genetic architecture has long been conceptualized within a dominant binary framework (Piet et al., 2026). At one end are “monogenic” epilepsies driven by rare, high-effect variants, particularly severe phenotypes such as developmental and epileptic encephalopathies (DEEs). At the other end are “complex” epilepsies shaped by the cumulative burden of numerous common variants with small effects (Allen et al., 2017; Epi25 Collaborative, 2019; Scheffer et al., 2024; International League Against Epilepsy Consortium on Complex Epilepsies, 2023; Koko et al., 2021). This framework has substantially advanced pathogenic gene discovery, molecular diagnosis, and mechanism-based treatment, thereby laying the foundation for clinical translation (Scheffer et al., 2024; Johannesen, 2023; EpiPM Consortium, 2015; Klein et al., 2024). However, with the parallel expansion of large-scale exome sequencing, copy number variant analysis, and genome-wide association studies, this “monogenic versus polygenic” stratification has increasingly revealed its limitations: although it captures the two ends of the variant-frequency and effect-size spectrum, it is insufficient to explain incomplete penetrance or the marked intrafamilial variability in age at onset, electroclinical phenotype, developmental outcome, and disease severity among individuals with the same molecular diagnosis in epilepsy (Piet et al., 2026; Campbell et al., 2022; Oliver et al., 2024).

Because much of the evidence reviewed below is derived from large-scale genetic studies that compare broad epilepsy subtypes, it is important to clarify how these categories are used in this Review. Consistent with the analytical framework adopted in studies such as Epi25, developmental and epileptic encephalopathies (DEEs), genetic generalized epilepsy (GGE), and non-acquired focal epilepsy (NAFE) are used as broad analytical groupings in genetic association studies (Epi25 Collaborative, 2019; Epi25 Collaborative, 2024). DEEs refer here to epilepsies in which developmental impairment reflects both the underlying etiology and the adverse effects of epileptic activity (Scheffer et al., 2017; Zuberi et al., 2022). GGE comprises generalized epilepsy syndromes characterized by generalized seizure types and typically generalized epileptiform discharges (Scheffer et al., 2017), whereas NAFE denotes a heterogeneous operational category of focal epilepsies without an identified acquired cause, rather than a single electroclinical syndrome (Epi25 Collaborative, 2019; Epi25 Collaborative, 2024). These categories are used as comparative groupings for describing patterns of genetic architecture and should not be interpreted as genetically discrete or mechanistically uniform entities. Although they differ in clinical presentation and in the relative contribution of different classes of genetic variation, their genetic risk profiles partly overlap across the allelic spectrum (Epi25 Collaborative, 2024).

Notably, the emergence of rare–common variant integration in epilepsy is not an isolated phenomenon, but rather part of a broader paradigm shift in neurogenetics. In neurodevelopmental disorders such as autism spectrum disorder, rare *de novo* variants, selected rare inherited variants, and pathogenic structural variants can exert moderate-to-large individual effects (Satterstrom et al., 2020; Zhou et al., 2022). However, common polygenic liability can also act additively with rare-variant effects to influence diagnostic thresholds, cognitive outcomes, and phenotypic severity (Weiner et al., 2017; Antaki et al., 2022; Huang et al., 2024). Similarly, in Parkinson’s disease, high-effect risk variants such as those in *GBA1* and *LRRK2* show incomplete penetrance, with disease risk and penetrance further modulated by genome-wide polygenic background and other genetic modifiers (Lai et al., 2021; Hassanin et al., 2025). Together, these cross-disorder findings suggest that both the probability of disease manifestation and the range of clinical expressivity may be influenced by the combined effects of high-effect variants and the broader genetic background (Antaki et al., 2022; Iwaki et al., 2020). This framework is particularly relevant to epilepsy, where seizure occurrence reflects dynamic state transitions in neural networks (Lopes da Silva et al., 2003; Jirsa et al., 2014). Genetic background may therefore influence not only overall epilepsy risk, but also seizure onset, electroclinical presentation, and disease severity (Oliver et al., 2024).

Within this cross-disorder integrative perspective, a central question in epilepsy becomes even more salient: why does the same pathogenic variant—or the same molecular diagnosis—not necessarily lead to the same clinical phenotype (Piet et al., 2026; Campbell et al., 2022; Oliver et al., 2024; Stevelink et al., 2026; Escayg et al., 2000; Martins Custodio et al., 2023)? In traditional clinical interpretation, penetrance is often summarized as a gene- or variant-associated estimate, whereas clinical severity is frequently attributed primarily to the implicated gene or to the functional consequence of the variant itself. However, growing evidence suggests that observed penetrance and clinical expressivity may both be shaped by the combined effects of the major-effect variant, polygenic background, variant functional class, regulatory context, and additional modifier loci (Campbell et al., 2022; Oliver et al., 2024; Stevelink et al., 2026; Wigdor et al., 2024; Castel et al., 2018). Accordingly, the observation of “same gene, different outcome” should not be dismissed as mere clinical contingency, but rather incorporated as a central issue within the genetic interpretive framework of epilepsy.

Against this background, this Review examines an integrative framework for rare and common genetic variation in epilepsy. We first summarize the genetic architecture of rare SNVs/indels, CNVs, and common variants across different epilepsy subtypes, with particular emphasis on their convergence and divergence at the levels of genes, gene sets, and biological pathways. We then discuss how polygenic background and other genetic modifiers may influence whether carriers of rare pathogenic variants or CNVs develop epilepsy and, among affected carriers, the severity and broader clinical expressivity of the phenotype. Finally, we consider the potential implications of this emerging framework for genetic counseling, variant interpretation, risk prediction, and precision medicine, as well as its current limitations.

## Reframing epilepsy genetics as a continuum of allelic risk

Although the dichotomous framework of “monogenic epilepsy” versus “complex epilepsy” has played an important role in disease-gene discovery and clinical genetic diagnosis, growing evidence suggests that it is better regarded as an empirical classification based on variant frequency, effect size, and study design, rather than as a representation of two entirely distinct disease mechanisms (Piet et al., 2026; Scheffer et al., 2024; Johannesen, 2023; EpiPM Consortium, 2015; Klein et al., 2024; Yano and Phitsanuwong, 2026). In traditional models, severe epilepsies such as DEEs are typically attributed to rare, high-effect variants, whereas more common epilepsies, including GGE and NAFE, are interpreted primarily in terms of common polygenic liability (Allen et al., 2017; Epi25 Collaborative, 2019; Scheffer et al., 2024; International League Against Epilepsy Consortium on Complex Epilepsies, 2023; Koko et al., 2021). However, recent studies have shown that different classes of genetic variation do not always exhibit clear boundaries across epilepsy subtypes; instead, they contribute to genetic risk in different proportions and with varying weights (Allen et al., 2017; Epi25 Collaborative, 2019; International League Against Epilepsy Consortium on Complex Epilepsies, 2023; Epi25 Collaborative, 2024; Niestroj et al., 2020).

First, common variants do not merely represent a nonspecific background risk. GWAS and PRS studies demonstrate a substantial common-variant contribution to GGE and its subtypes, with subtype-specific risk loci and measurable polygenic liability (International League Against Epilepsy Consortium on Complex Epilepsies, 2023; Koko et al., 2021; Vorderwülbecke et al., 2022; Heyne et al., 2024; Kubota et al., 2025; Karadag et al., 2026).

Second, the contribution of rare SNVs/indels is not confined to epilepsies traditionally regarded as “monogenic”. Large-scale sequencing studies such as Epi25 have shown that DEEs, GGE, and NAFE exhibit both syndrome-specific burdens of rare variation and shared risk genes, gene sets, and biological processes (Allen et al., 2017; Epi25 Collaborative, 2019). Accordingly, the contribution of rare variants to disease liability and clinical expression should not be inferred solely from whether they are classified as “pathogenic”, but should also be considered in relation to gene dosage sensitivity, spatiotemporal developmental expression, functional consequences, and the individual’s broader genetic background (Oliver et al., 2024; Richards et al., 2015; Chi and Kiskinis, 2024).

Third, structural variation further blurs the boundary between monogenic and complex epilepsy. Multiple recurrent CNV loci have been associated with epilepsy and neurodevelopmental disorders and often show incomplete penetrance and variable expressivity (Montanucci et al., 2023; Moreau et al., 2022). The burden and type of CNVs vary across epilepsy subtypes: larger CNVs affecting multiple dosage-sensitive genes are more commonly observed in DEEs and epilepsies with neurodevelopmental comorbidities, whereas smaller or locus-specific CNVs also contribute to risk in GGE, NAFE, and familial common epilepsies (Niestroj et al., 2020; Montanucci et al., 2023; Moreau et al., 2022; Epi4K Consortium, 2024). Therefore, CNVs should be regarded as an important structural layer of risk that spans the continuum between rare high-effect variants and complex genetic liability.

Finally, at the individual and family levels, polygenic background provides a plausible explanatory layer for incomplete penetrance and phenotypic heterogeneity. Even among individuals carrying established or presumed rare variants of large effect, epilepsy may or may not occur. Among affected carriers, age at onset, epilepsy type, cognitive outcome, and clinical severity may also vary substantially (Oliver et al., 2024; Escayg et al., 2000; Dibbens et al., 2013; Colosimo et al., 2007; Dang et al., 2026). In DEEs and other epilepsies associated with intellectual disability, common polygenic liability for epilepsy was evident in cases both with and without an identified rare deleterious variant of major effect (Campbell et al., 2022). In genetic epilepsy with febrile seizures plus (GEFS+) families, higher epilepsy PRS is associated with both disease manifestation and greater phenotypic severity (Oliver et al., 2024), while analyses of mixed generalized/focal epilepsy families and monozygotic twins provide additional evidence that common variant burden contributes to familial and individual differences in epilepsy liability (Dang et al., 2026; Ellis et al., 2025). Longitudinal population data further suggest that this contribution has life-course relevance: using electronic health records from more than 700,000 individuals, Heyne et al. found that each standard-deviation increase in PRS_GGE was associated with a hazard ratio of 1.73 for lifetime GGE and approximately 1.5 during the 10 years following an unspecified seizure event (Heyne et al., 2024). Although these findings do not establish gene-specific modification of rare-variant penetrance, they indicate that common variant burden contributes to epilepsy susceptibility across population, family, and presumed monogenic contexts (Stevelink et al., 2026).

Taken together, the genetic architecture of epilepsy is better understood as a continuum spanning allele frequency, effect size, and variant class, rather than as two discrete etiologic categories of “monogenic” and “polygenic” disease. Within this framework, severe phenotypes such as DEEs are typically more strongly driven by ultra-rare, large-effect variants, whereas more common epilepsies such as GGE more clearly reflect common polygenic liability; however, there is no absolute boundary between the two. The biological basis of the overlap among these variant classes is considered in the following section.

## Convergence across the allelic spectrum

An important advance in epilepsy genetics lies not only in identifying additional risk loci, but also in showing that variants across the allele-frequency and effect-size spectrum may converge on partially shared genes and biological pathways contributing to epilepsy susceptibility (Koko et al., 2021; Epi25 Collaborative, 2024; Delahaye-Duriez et al., 2016). Here, “convergence” does not primarily refer to the simple recurrent involvement of the same locus by different classes of variants; rather, it is more clearly reflected in partial overlap at the levels of candidate gene prioritization, functional gene sets, cell-type specificity, and synaptic–excitability networks (Epi25 Collaborative, 2024; Montanucci et al., 2023).

At the gene level, GWAS candidate genes overlap with known monogenic epilepsy genes and antiseizure medication targets, suggesting that common variants and rare high-effect variants may implicate the same genes or closely related pathogenic networks (International League Against Epilepsy Consortium on Complex Epilepsies, 2023; Mastrangelo et al., 2025). The Epi25 study further showed that some GGE GWAS candidate genes are also supported by ultra-rare variant burden analyses, extending cross-frequency validation from locus proximity to gene-level evidence (Epi25 Collaborative, 2024). However, such gene-level overlap is observed only for a subset of implicated genes, and convergence across the allelic spectrum is often more apparent at the level of shared functional modules and biological networks. This many-to-one convergence may help explain why genetically distinct variants can produce overlapping electroclinical phenotypes, thereby limiting the phenotypic specificity and individual-level predictive value of any single variant.

At the pathway and cell-type levels, this functional convergence becomes even more apparent. Rare high-effect variants directly implicate voltage-gated ion channels, GABA_A receptor subunits, and synapse-related genes, whereas common variant signals are likewise enriched in processes related to neuronal excitability, synaptic transmission, and excitatory–inhibitory balance (International League Against Epilepsy Consortium on Complex Epilepsies, 2023; Chi and Kiskinis, 2024; Specchio et al., 2024; Absalom et al., 2022). Biological distinctions nevertheless persist across epilepsy subtypes: in GGE, common variant signals more prominently point to synaptic functions in excitatory and inhibitory neurons as well as gene expression programs associated with the postnatal neocortex (International League Against Epilepsy Consortium on Complex Epilepsies, 2023; Epi25 Collaborative, 2024), whereas in DEE, ultra-rare damaging variants place greater emphasis on early neurodevelopment and the vulnerability of genes related to inhibitory neurons (Epi25 Collaborative, 2019; Koko et al., 2021). Thus, the points of convergence across the allelic spectrum do not lie in any single repeatedly implicated “shared gene”, but rather in interacting biological axes involving transcriptional programs, neuronal excitability, synaptic stability, and network seizure threshold (International League Against Epilepsy Consortium on Complex Epilepsies, 2023; Epi25 Collaborative, 2024; Dang et al., 2026; Delahaye-Duriez et al., 2016). Although these pathways are not exclusive to epilepsy, their direct relationship to electrophysiological dysfunction, seizure generation, and targets of antiseizure medications gives them particular relevance in epilepsy genetics.

**FIGURE 1:**
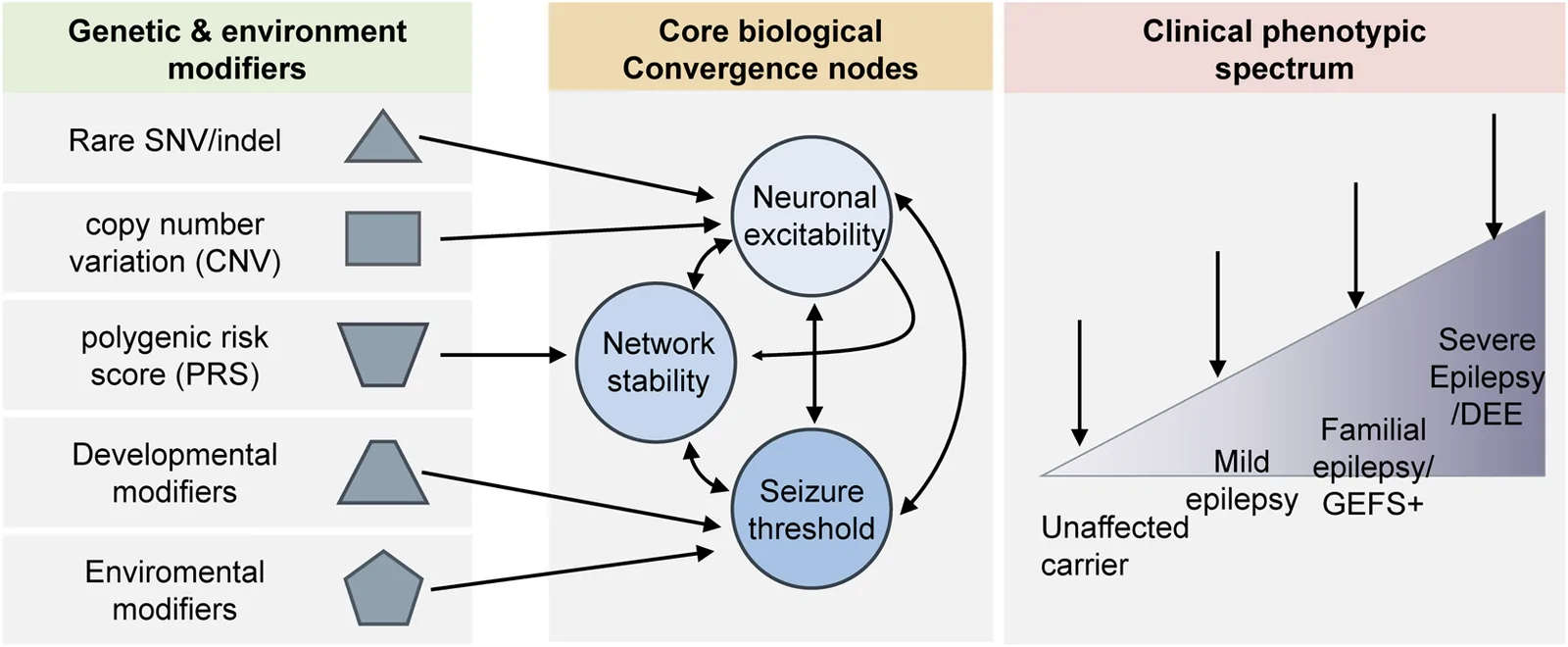
Genetic modifiers of epilepsy penetrance and clinical expression. Rare SNVs/indels, copy number variants (CNVs), polygenic risk burden, often quantified by polygenic risk scores (PRS), and developmental and environmental modifiers may influence epilepsy susceptibility through distinct genetic and non-genetic pathways. These factors do not map simply onto independent disease categories, but instead partially converge on core biological nodes such as neuronal excitability, network stability, and seizure threshold. Different combinations of risk burden and modifying factors may determine whether an individual crosses the threshold for disease manifestation and may also influence the degree of clinical expression, thereby giving rise to a phenotypic continuum ranging from unaffected carriers and mild epilepsy to familial epilepsy/GEFS+, severe epilepsy, and developmental and epileptic encephalopathy (DEE). This figure presents a conceptual model intended to summarize the central themes of this review, including integration across the allele-frequency spectrum, modification of penetrance, and continuity of clinical phenotypes.

Studies of structural variation further reinforce this form of network bridging at the level of gene dosage. Research on familial GGE and NAFE suggests that CNVs can contribute to risk in common familial epilepsies, including potentially pathogenic variants that disrupt known epilepsy genes or involve recurrent epilepsy-associated loci (Epi4K Consortium, 2024). Large-scale CNV analyses have also shown that, although CNVs do not necessarily overlap directly with GWAS loci, they can link direct disruption of core epilepsy genes to broader disturbances in neurodevelopmental and synaptic networks by altering the dosage of multiple genes (Karadag et al., 2026; Montanucci et al., 2023). Thus, CNVs are not simply an intermediate class between rare and common variants; rather, they represent an important category of variation in the genetic architecture of epilepsy by acting through gene dosage effects, large-scale regulatory alterations, and network-level perturbations. Together, these findings support a network-level rather than strictly gene-by-gene model of convergence across the allelic spectrum (Figure 1).

## Hierarchical modification of penetrance and expressivity

Existing evidence indicates that disease manifestation in a carrier is not solely a fixed property of a rare pathogenic variant. At the group level, the observed penetrance of a variant may instead reflect the cumulative effects of multilayered genetic burden (Stevelink et al., 2026). In this context, polygenic background is no longer merely a population-level statistical signal; rather, it may contribute to shaping the clinical outcomes of individuals carrying rare variants (Oliver et al., 2024; Dinneen et al., 2024; De Sainte Agathe and Leguern, 2025).

**FIGURE 2:**
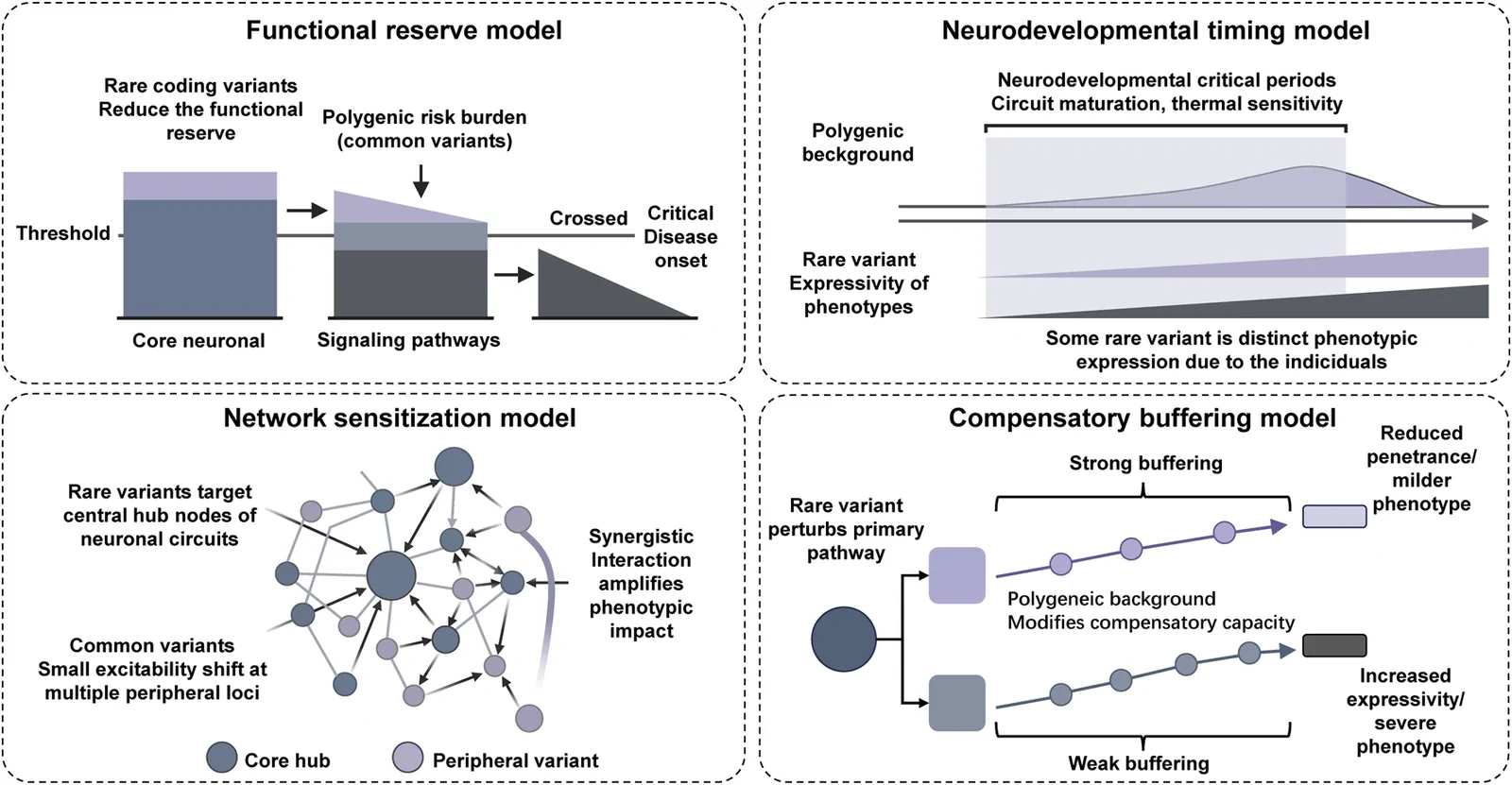
Polygenic modification of rare-variant penetrance in epilepsy. Polygenic background may influence the clinical consequences of rare pathogenic variants or CNVs through several non-mutually exclusive mechanisms. The functional reserve model proposes that common-variant burden may reduce neurological reserve, making an individual more likely to cross the disease threshold. The neurodevelopmental timing model emphasizes that polygenic background may alter circuit maturation and susceptibility during critical developmental windows. The network sensitization model suggests that disruption of core hub nodes by rare variants may interact with the cumulative small effects of multiple peripheral common variants, leading to synergistic amplification. The compensatory buffering model further suggests that polygenic background may modulate an individual’s capacity to buffer the effects of a major-effect variant, thereby influencing penetrance, severity, and phenotypic expression. This figure presents a conceptual model intended to integrate possible pathways through which rare variants, CNVs, and common variants jointly shape epilepsy penetrance and clinical heterogeneity.

Conceptually, the modifying effects of polygenic background on the penetrance and clinical expressivity of rare pathogenic variants are unlikely to operate through a single pathway, but may involve several complementary layers that can be summarized into four non-mutually exclusive models (Figure 2). These conceptual models organize current association findings but are not experimentally established mechanisms in epilepsy. First, polygenic burden may reduce the functional reserve of the nervous system, rendering individuals more susceptible to being pushed across the disease threshold by a rare variant (Huang et al., 2024; Kingdom et al., 2024). Second, polygenic background may alter circuit maturation and vulnerability during critical windows of neurodevelopment, thereby influencing age at onset and phenotypic expression (Campbell et al., 2022; Epi25 Collaborative, 2024). Third, a rare variant affecting a key node within a neuronal network and common variants exerting small, distributed effects on neuronal excitability may act additively or interactively to increase network susceptibility (Oliver et al., 2024; Epi25 Collaborative, 2024). Fourth, polygenic background may affect putative buffering and feedback mechanisms within gene-expression and neuronal networks, thereby affecting whether a carrier remains unaffected or develops a mild or more severe clinical phenotype (Martins Custodio et al., 2023; Kingdom et al., 2024).

It is important to further emphasize that such modifying effects are not synonymous with the classical PRS alone (Rizvi et al., 2025). Clinical expressivity includes seizure type, age at onset, developmental outcome, and severity; treatment response is distinct, although both may be influenced by additional rare variants, oligogenic interactions, and regulatory background (Martins Custodio et al., 2023). Additional rare variants within the same biological pathway may amplify the phenotypic consequences of a primary high-effect variant (Kingdom et al., 2024), whereas cis- or trans-regulatory variants may modify the effects of coding variants by altering the expression levels of target genes (Wigdor et al., 2024; Castel et al., 2018). Accordingly, penetrance and expressivity are jointly shaped by the primary variant, polygenic background, additional modifier variants, developmental context, and environmental exposures, rather than being direct reflections of any single genetic metric (Stevelink et al., 2026; Kingdom et al., 2024; Jensen et al., 2025; Kingdom and Wright, 2022).

Gene-specific observations provide concrete illustrations of this continuous and multilayered genetic architecture, rather than supporting a simple division between monogenic and polygenic epilepsies. Within the *SCN1A*-related epilepsy spectrum, loss-of-function variants are most commonly associated with phenotypes ranging from febrile seizures and GEFS+ to Dravet syndrome, whereas gain-of-function variants are associated with additional, mechanistically distinct phenotypes (Escayg et al., 2000; Colosimo et al., 2007; Knox et al., 2025; Brunklaus et al., 2022a; Brunklaus et al., 2022b). Although variant function is an important determinant of phenotypic liability, it is neither sufficient nor uniformly predictive of the final clinical outcome. Additional rare variants in epilepsy-associated genes and blended molecular diagnoses have been identified in some individuals with Dravet syndrome (Martins Custodio et al., 2023). Family-based evidence in GEFS+ further suggests that common polygenic burden can modify disease penetrance and phenotypic severity among relatives sharing a high-effect rare variant (Oliver et al., 2024). In variant-specific analyses, epilepsy PRS showed a strong association with phenotypic expression among relatives carrying *GABRG2* p.Arg82Gln, whereas no significant association was detected among relatives carrying *SCN1B* p.Cys121Trp (Oliver et al., 2024). These findings suggest that susceptibility to polygenic modification may differ between rare variants and their associated genetic contexts.

Other epilepsy genes illustrate additional layers of the same continuum. In *DEPDC5*-related epilepsy, germline pathogenic variants show marked incomplete penetrance and intrafamilial variability, while brain-restricted somatic second-hit variants provide a mechanism through which additional postzygotic burden may shift an individual toward focal cortical dysplasia and more severe focal epilepsy (Ribierre et al., 2018; Alsayed et al., 2025). In several other genes, including *SCN2A*, *SCN8A*, *KCNQ2*, and *GABRB2*/*GABRB3*, current genotype–phenotype evidence more directly supports a major contribution from the functional direction and magnitude of the primary variant, including gain- and loss-of-function effects associated with differences in seizure onset, epilepsy phenotype, neurodevelopmental outcome, and treatment response (Absalom et al., 2022; Brunklaus et al., 2022a; Brunklaus et al., 2022b; Thompson et al., 2023; Mohammadi et al., 2024; Hack et al., 2024; Yang et al., 2023). *STXBP1*-related disorders show substantial variation in seizure trajectories and developmental outcomes, although much of this heterogeneity remains unexplained (Xian et al., 2023; Xian et al., 2022). These examples should not be interpreted as discrete gene-specific models. Rather, they illustrate that the relative contributions of a high-effect variant, common polygenic burden, additional rare variants, somatic events, and developmental or environmental context vary across individuals and molecular etiologies. The resulting clinical outcome may therefore reflect an underlying continuous liability rather than the direct and fixed consequence of a single pathogenic variant.

For CNVs and other dosage-altering structural variants, the issue of penetrance modification is equally relevant. Large-scale studies have shown that neurodevelopmental disorder-associated CNVs (ND-CNVs) commonly exhibit incomplete penetrance and substantial phenotypic variability, and that estimates of their penetrance are influenced by cohort source, phenotype definition, and ascertainment bias (Montanucci et al., 2023; Dinneen et al., 2022; Goh et al., 2025). Among carriers of ND-CNVs, cognitive and neurodevelopmental outcomes may also be influenced by polygenic scores, additional rare variants, and other secondary variants (Dinneen et al., 2024; Jensen et al., 2025). Thus, epilepsy-associated CNVs may confer baseline risk, while penetrance and expressivity may also be influenced by gene dosage, CNV size, genetic background, and environmental factors (Dinneen et al., 2024; Jensen et al., 2025). Nevertheless, compared with rare SNVs/indels, direct family-based evidence on how common variant burden or additional rare variants modify the penetrance of such structural variants remains relatively limited.

## A probabilistic framework for clinical translation

**FIGURE 3:**
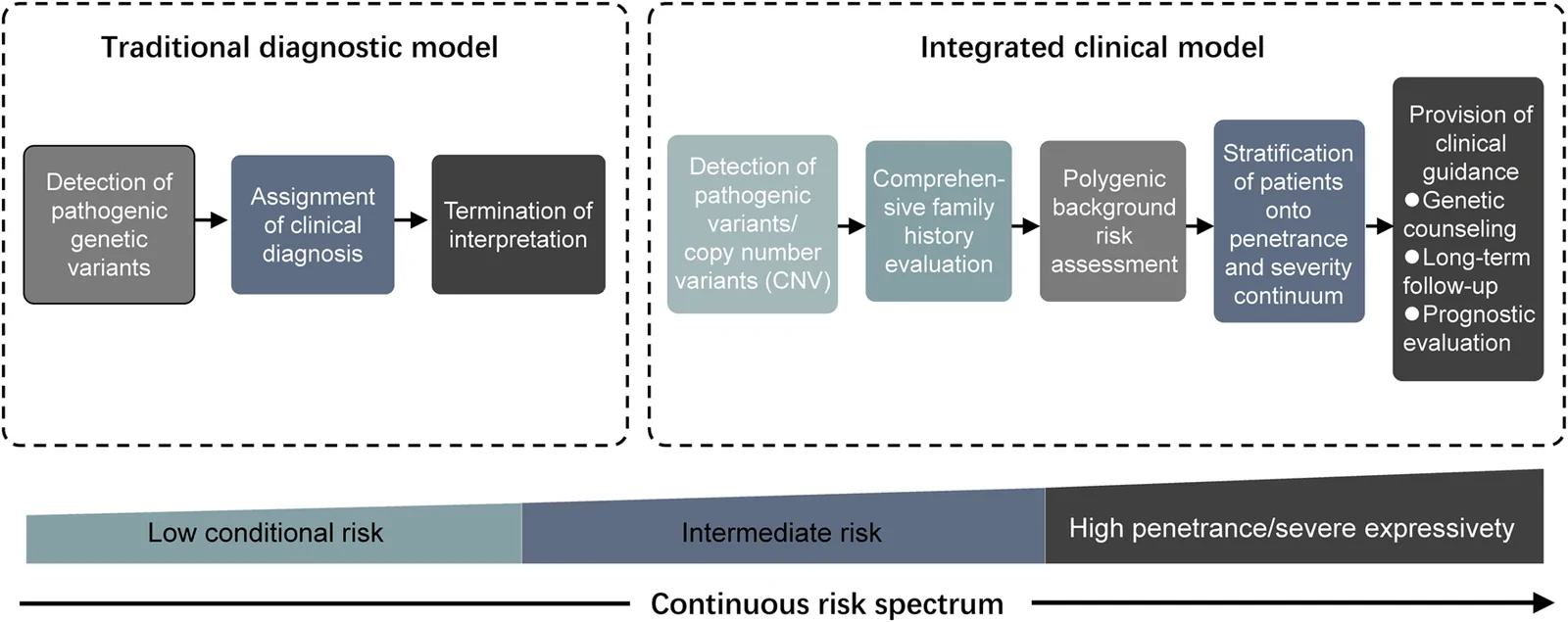
Integrated clinical interpretation in epilepsy genetics. The traditional diagnostic approach primarily focuses on the detection of pathogenic genetic variants, assignment of a clinical diagnosis, and termination of interpretation. In contrast, the integrated clinical model incorporates comprehensive family history evaluation and assessment of polygenic background risk, enabling the stratification of patients along a continuum of penetrance and clinical severity. This approach supports the provision of personalized clinical guidance, including genetic counseling, long-term follow-up, and prognostic evaluation. The continuous risk spectrum depicted below illustrates the range from low conditional risk to high penetrance and severe expressivity.

If the studies discussed above have reshaped our understanding of the genetic architecture of epilepsy, their clinical translational value lies in providing a more refined interpretive framework. This framework does not diminish the importance of molecular diagnosis; rather, it redefines its clinical meaning. In severe epilepsies such as developmental and epileptic encephalopathies, sequencing can identify a potential underlying cause in a substantial proportion of patients (Boonsimma et al., 2023). In an observational study, clinical management was modified in 49.8% of patients following a genetic diagnosis, and improvement was reported in 74.9% of those with available follow-up data (McKnight et al., 2022). However, the absence of a control group, the potential contribution of concurrent clinical changes, and the fact that not all management modifications were necessarily variant-specific preclude attributing these outcomes directly to the genetic diagnosis. Molecular diagnosis may therefore provide clinically useful etiological and management information in selected cases, but its independent effect on patient outcomes remains insufficiently established. It should be interpreted alongside family history, variant function, broader genetic background, available penetrance estimates, and expected clinical expressivity (Figure 3).

At the level of testing strategy, this framework further underscores the need for subtype-specific genetic evaluation. For DEEs and other early-onset severe epilepsies, rare high-effect variants, *de novo* variants, and larger CNVs remain the primary targets of genetic testing (Allen et al., 2013; Mefford et al., 2011). By contrast, in more common subtypes with a more diffuse genetic architecture, such as GGE, common variant burden may be more informative for characterizing an individual’s background susceptibility (International League Against Epilepsy Consortium on Complex Epilepsies, 2023; The International League Against Epilepsy, 2018; Leu et al., 2019). Accordingly, sequencing-based rare-variant analysis and CNV/structural-variant assessment should be selected according to phenotype and age at onset (Krey et al., 2022; Sheidley et al., 2022). Polygenic risk metrics may provide complementary information in selected common epilepsy subtypes, but their clinical utility requires prospective validation (Heyne et al., 2024; Kubota et al., 2025; Abu-El-Haija et al., 2023). At present, however, PRS is better suited as a tool for risk stratification and interpretation of genetic background than as an independent diagnostic criterion. PRS is not currently incorporated into the ACMG/AMP criteria for VUS classification and should not substitute for phenotype matching, familial segregation, functional evidence, population frequency data, or periodic reanalysis (Richards et al., 2015; Abu-El-Haija et al., 2023).

Genetic counseling and risk prediction need to move beyond a binary interpretation based solely on whether a pathogenic variant is present, toward a probabilistic model that also considers the potential influence of broader genetic background. Family- and population-based studies indicate that observed penetrance may differ across carrier groups and phenotypic severity may vary among affected carriers, although current estimates remain insufficient for individualized prediction (Campbell et al., 2022; Oliver et al., 2024; Stevelink et al., 2026; Ellis et al., 2025; McTague et al., 2022). Clinical interpretation should consider not only variant presence, but also penetrance, expressivity, and uncertainty within the relevant genetic, familial, and clinical context. GGE currently provides one of the clearest examples of subtype-specific PRS association in epilepsy, although current scores are not sufficiently validated for routine clinical risk stratification (Heyne et al., 2024). However, the discriminative performance of current PRSs remains limited; their explanatory value is weaker for subtypes such as focal epilepsy, and their clinical application is further constrained by factors including cross-ancestry portability, standardized calculation, and communication of results (Kubota et al., 2025; Wang S. et al., 2025; Xiang et al., 2024). A similar consideration applies to recurrent neurodevelopment-associated CNVs. Although multiple epilepsy-related loci have been identified, their penetrance and phenotypic variability still cannot be fully explained by the CNV itself. At present, direct clinical evidence that PRS or additional rare variants modify the penetrance of epilepsy-associated CNVs remains limited (Epi4K Consortium, 2024; Goh et al., 2025; Goh et al., 2026).

Precision medicine likewise needs to move beyond a framework driven by single-gene labels toward one that integrates functional stratification with multilayered genetic background (Oliver et al., 2024; Wolff et al., 2017). However, trials in tuberous sclerosis complex show that pathway- or mechanism-directed treatment may improve specific seizure outcomes without parallel benefits across cognition, development, and all seizure types (French et al., 2016; Bebin et al., 2023). Among patients with the same molecular diagnosis, age at onset, disease course, severity, and cognitive outcome may be influenced by variant function and broader genetic background. Treatment response may also vary within the same molecular diagnosis, although direct evidence that epilepsy PRS modifies treatment response remains limited (Oliver et al., 2024; Martins Custodio et al., 2023; Knox et al., 2025; Brunklaus et al., 2022a; Brunklaus et al., 2022b). Accordingly, when a clear relationship among genetic variation, functional effects, and treatment response has been established, precision therapy requires more than identifying the affected gene. It also requires determining how the specific variant influences protein function, neural network excitability, and drug sensitivity. In channelopathies and receptor-related epilepsies, gain-of-function and loss-of-function variants may produce opposite physiological consequences and therefore call for different therapeutic strategies (Absalom et al., 2022; Brunklaus et al., 2022a; Brunklaus et al., 2022b; Mohammadi et al., 2024). Studies of *SCN1A*, *SCN2A*, *SCN8A*, *KCNQ2*, and *GABRB2* provide a mechanistic rationale for functionally stratified treatment (Knox et al., 2025; Thompson et al., 2023; Mohammadi et al., 2024; Hack et al., 2024; Yang et al., 2023), although direct prospective evidence that such stratification improves clinical outcomes remains limited. The limited or variable effects of some gene- and pathway-directed treatments are also consistent with a systems-level model in which correction of a single molecular component may be insufficient to normalize an emergent phenotype shaped by broader genomic, developmental, and neural-network interactions.

Overall, the clinical translation of epilepsy genetics is shifting from a deterministic, label-based paradigm toward an integrated model that is probabilistic, context-dependent, and functionally informed. Within this framework, molecular diagnosis remains the clinical anchor, but its significance must be interpreted in conjunction with penetrance, polygenic background, CNVs and additional rare variants, variant function, and the patient’s phenotype.

## Current limitations and future directions

Although the framework integrating rare and common variation is reshaping epilepsy genetics, the current evidence remains uneven across molecular etiologies, epilepsy subtypes, study designs, and clinical outcomes (Piet et al., 2026; Kubota et al., 2025). Large-scale genomic association studies provide relatively strong support for shared and subtype-specific genetic architectures (International League Against Epilepsy Consortium on Complex Epilepsies, 2023; The International League Against Epilepsy, 2018), whereas direct mechanistic evidence and evidence of prospective clinical utility remain more limited. In particular, although polygenic background has been associated with disease manifestation or phenotypic severity in selected syndromes and families, comparable gene- or variant-specific effects on penetrance or expressivity have not been consistently demonstrated across most epilepsies associated with rare pathogenic variants (Oliver et al., 2024; Martins Custodio et al., 2023). Moreover, modifiers of penetrance may differ from those affecting expressivity or treatment response (Stevelink et al., 2026; Kingdom and Wright, 2022). First, existing GWAS, PRS, and rare-variant studies have been conducted predominantly in populations of European ancestry, limiting the transferability, calibration, and clinical equity of risk models in other ancestral groups (Heyne et al., 2024; Kubota et al., 2025; Xiang et al., 2024). Second, the current genetic landscape remains incomplete: the roles of noncoding variants, complex structural variants, somatic mosaicism, and epigenetic regulation in epilepsy have yet to be systematically characterized (LaFlamme et al., 2024; Chang et al., 2025; Henry et al., 2025; Baldassari et al., 2025). Third, many candidate variants, especially missense variants, still lack reproducible functional validation, creating a substantial gap between pathogenicity assessment, functional stratification, and therapeutic inference (Montanucci et al., 2024; Heyne et al., 2020). Finally, heterogeneity across studies in sample ascertainment, phenotype definition, variant filtering, statistical modeling, and functional assays limits cross-study integration and the standardization of clinical translation (Moloney and Delanty, 2025). Such heterogeneity may also partly explain why findings regarding genetic modification have not always been consistent across studies.

Future research should prioritize the development of large, deeply phenotyped, and multi-ancestry cohorts to enhance the generalizability of PRS and combined rare–common variant models (Abu-El-Haija et al., 2023; Han et al., 2025). Gene- and syndrome-specific studies should also include both affected and unaffected carriers of pathogenic variants to enable more direct investigation of incomplete penetrance and variable expressivity. Whole-genome sequencing, transcriptomic, proteomic, epigenomic, and single-cell data should be integrated to link genetic variation with cellular function, neural network alterations, and clinical outcome (Baldassari et al., 2025; Wang N. N. et al., 2025; Zheng et al., 2025). High-throughput and reproducible functional validation platforms are also needed to systematically characterize gain- and loss-of-function effects, dosage sensitivity, and drug-response profiles of epilepsy-associated variants (Vanoye et al., 2022). In parallel, future prospective studies should jointly evaluate rare variants, CNVs, PRS, and functional evidence, building on current diagnostic practice and variant-reanalysis frameworks (Krey et al., 2022; Chourasia et al., 2024). Longitudinal electroclinical phenotyping is needed to distinguish modifiers of penetrance, expressivity, and treatment response (Oliver et al., 2024; Palmer et al., 2021). Only by creating a closed loop of evidence—from statistical association through functional mechanism to clinical utility—can epilepsy genetics advance from variant discovery toward interpretable, predictive, and actionable precision medicine.

## Conclusion

In summary, epilepsy genetic risk is best understood as a multilayered architecture in which rare high-effect variants, structural variation, common polygenic liability, and other modifiers contribute in different proportions across molecular etiologies and epilepsy subtypes. These components may jointly shape disease susceptibility, penetrance, and clinical expressivity, but their relative contributions remain incompletely resolved.

Clinically, molecular diagnosis should remain central but must be interpreted within the broader genetic, familial, functional, and phenotypic context. At present, integrated rare–common models are more mature as explanatory frameworks than as tools for routine individualized prediction or treatment selection, and their clinical use will require prospective validation.
